# Eight Weeks of Aerobic Interval Training Improves Psychomotor Function in Patients with Parkinson’s Disease—Randomized Controlled Trial

**DOI:** 10.3390/ijerph16050880

**Published:** 2019-03-11

**Authors:** Jarosław Marusiak, Beth E. Fisher, Anna Jaskólska, Krzysztof Słotwiński, Sławomir Budrewicz, Magdalena Koszewicz, Katarzyna Kisiel-Sajewicz, Bartosz Kamiński, Artur Jaskólski

**Affiliations:** 1 Department of Kinesiology, Faculty of Physiotherapy, University School of Physical Education, Al. I.J. Paderewskiego 35, Building P4, 51-612 Wroclaw, Poland; anjaskol@gmail.com (A.J.); katarzyna.kisiel-sajewicz@awf.wroc.pl (K.K.-S.); bartekaminski1991@gmail.com (B.K.); artur.jaskolski@awf.wroc.pl (A.J.); 2 Division of Biokinesiology and Physical Therapy and Department of Neurology, University of Southern California, Los Angeles, CA 90033, USA; bfisher@usc.edu; 3 Department of Neurology, Medical University of Wroclaw, 50-566 Wroclaw, Poland; k.slot@gazeta.pl (K.S.); s.budrewicz@wp.pl (S.B.); magdalena.koszewicz@umed.wroc.pl (M.K.)

**Keywords:** Parkinson’s disease, aerobic interval training, neuroplasticity, bimanual motor control, executive function

## Abstract

Background: This study examined the generalized effects of cycle ergometer aerobic interval training (AIT) on psychomotor behaviors in individuals with Parkinson’s disease (PD), including bimanual motor control, cognitive function, and neurological motor and non-motor parkinsonian signs. Methods: Twenty mild to moderate PD patients were randomly allocated to the following groups: (1) trained group (PD-TR, *n* = 10), which besides receiving usual care, underwent an 8-week moderate intensity AIT program; or (2) control group (PD-CO, *n* = 10) which received usual care, including participation in conventional physical therapy. Both groups were tested before and after the 8-week AIT program period with the following assessments: (1) laboratory analyses of bimanual motor control, (2) psychological evaluation of cognitive function, and (3) an evaluation of neurological parkinsonian signs. Results: The PD-TR group exhibited improved (1) bimanual motor control, reflected by a decreased time (*p* = 0.013) and increased rate of grip force development (*p* = 0.013) in the manipulating hand and a decreased time delay between grip force initiation in the manipulating and stabilizing hand (*p* = 0.020); (2) executive function, reflected by decreased performance time in part II of the Stroop Test (*p* = 0.007); and (3) neurological parkinsonian signs, reflected by an amelioration of upper-extremity bradykinesia (*p* = 0.015) and improvement in daily life manual functions (*p* = 0.004), mood, and intellectual function (*p* = 0.005). Conclusions: Following an 8-week moderate intensity AIT program, patients with PD exhibited improved psychomotor behaviors, reflected by bimanual motor control, executive function, and neurological parkinsonian signs.

## 1. Introduction

Parkinson’s disease (PD) is a progressive neurodegenerative condition characterized by dopamine (DA) depletion due to the degeneration of dopaminergic neurons in the substantia nigra pars compacta [1]. PD impacts motor and cognitive function secondary to the shared DA-dependent neural structures [2,3,4,5,6]. The gold standard treatment for Parkinson’s disease is DA replacement pharmacotherapy, which does not stop the neurodegenerative process and has negative side effects [7]. According to previous reports on animal models of parkinsonism, high-intensity exercise induces both behavioral and neuroplastic changes, including an improvement in DA terminal function in the central nervous system (CNS) [8,9,10,11,12,13]. These findings have been partly translated to human studies [14,15,16,17,18,19,20,21,22,23], potentially revealing the basic mechanisms underlying numerous reports of exercise-induced motor and cognitive improvements in patients with PD [14,15,16,17,18,19,20,21,23]. Most human studies [14,15,16,17,21,22] have applied continuous intensive training. However, in previous studies [24,25,26], interval training has been shown to be more enjoyable and effective than continuous exercise programs in a non-PD population (healthy adults [24,25] and adult cardiological patients [26]). According to Zoladz et al. [20] and Marusiak et al. [23], moderate intensity aerobic interval training (AIT) has the potential to induce beneficial neuroplastic changes in the CNS in trained individuals with PD. In these studies [20,23], the AIT-induced increase in brain derived neurotrophic factor (BDNF) was demonstrated. However, the beneficial effects of moderate intensity AIT performed on a cycloergometer on symptoms and specific cognitive (executive function) and motor (automatic bimanual motor control) aspects of psychomotor behaviors in patients with PD have not been established. Therefore, in the current study, our primary outcomes were automatic bimanual motor control measures and the psychological evaluation of cognitive functions. Thus, in our PD patients, we tested performance of an anti-phase bimanual motor coordination task, because this task has been previously reported to be strongly dependent on dopaminergic function [2,3,4,5,6]. For the psychological cognitive function assessments, we primarily chose executive function tests, i.e., the trail making test (TMT) and the Stroop Test (ST) [27,28,29,30,31], because performance on these tests is related to DA-dependent striatocortical neuronal networks and executive function deficits in PD. These deficits include working memory, planning, and inhibitory control as well as episodic memory and visuospatial functioning [32]. These executive function deficits appear years before the cardinal motor symptoms allow clinical diagnosis (prodromal stage of disease) and worsen in PD dementia [32]. Our secondary outcome measures were neurological signs of PD, with a primary focus on aspects of the unified Parkinson’s disease rating scale that concern precise one-handed and bimanual motor functions as well as cognitive aspects of non-motor symptoms. These neurological PD signs were chosen since they are related to the DA-dependent subcortical and cortical neural structures [2,3,4,5,6]. Thus, our goal was to study the generalized effects of moderate intensity AIT performed on a cycle ergometer by the lower extremities on DA-dependent psychomotor behaviors [2,3,4,5,6,27,28,29,30,31], including upper extremity automatic bimanual motor control, executive function as well as neurological motor and non-motor signs of PD. Our general hypothesis was that moderate intensity AIT could result in psychomotor behavioral improvement in trained individuals with PD, which would be reflected in the above mentioned primary and secondary outcome measures.

## 2. Materials and Methods

### 2.1. Subjects

Patients with idiopathic PD were recruited from the Clinic of Neurology at Medical University of Wroclaw (CN MUW) and from the Circle of Friends of People with Parkinson’s Disease in Wroclaw. The inclusion criteria were age >55 years-old, diagnosis of idiopathic PD, and modified Hoehn and Yahr stages between 1.5 and 3. The exclusion criteria were (1) presence of other neurological disorders, (2) any cardiovascular or respiratory system restrictions and/or motor deficits that could limit performance in high-speed pedaling on a cycle ergometer or in conventional physical therapy, and (3) practicing any regular physical activity except for conventional physical therapy for PD. Compliance with the study criteria was assessed by the patients’ medical records, a physician’s interview, and a clinical neurological examination. The subjects were informed of the aim of the study and provided their written consent prior to the study, which was approved by the local Ethics Committee of the University School of Physical Education in Wroclaw (project identification code: 0247/p01/2010/70) and in accordance with the Code of Ethics included in the Helsinki Declaration. Our study protocol was registered as a clinical trial (ClinicalTrials.gov identifier: NCT03753503).

**Figure 1:**
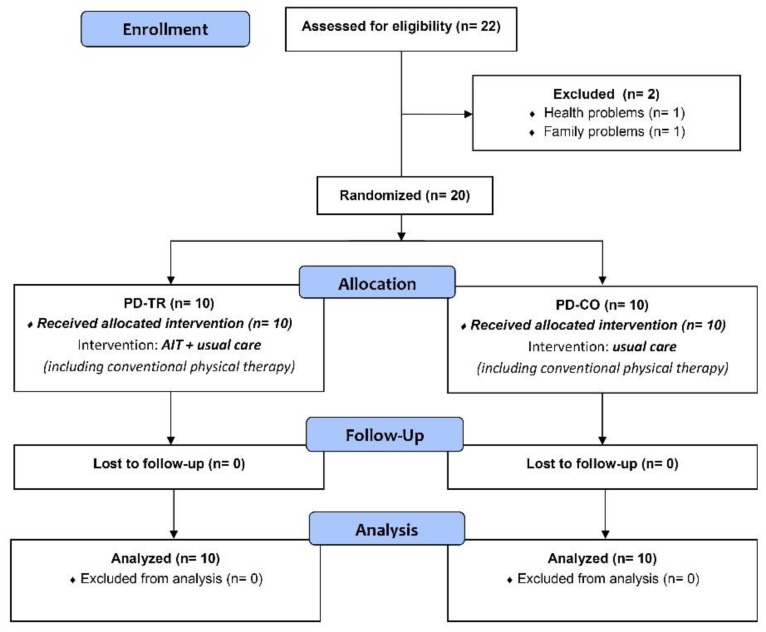
Flow diagram.

Twenty-two patients with PD were initially enrolled to participate in our study, and two patients were excluded due to health problems (*n* = 1) and family problems (*n* = 1) (Figure 1). According to the Hoehn and Yahr scale (H&Y) [33], all PD patients were mildly to moderately affected (H&Y score of 1.5–3.0) (Table 1). The 20 patients were randomly allocated into one of two groups: the training group (PD-TR, *n* = 10) or the control group (PD-CO, *n* = 10) (Figure 1) (Table 1). Based on previously published data [20,21,23], we statistically estimated that a sample size of 10 per group would be sufficient to test our hypotheses and obtain statistically significant results. The whole block type randomization process was carried out by the authors of this study using sequentially numbered sealed envelopes. Opaque envelopes were used to conceal allocation. Patients randomized to the PD-CO group were asked to maintain their usual lifestyle during the study period and received usual care including participation in conventional physical therapy that did not involve moderate or high intensity aerobic interval training on a cycle ergometer or treadmill. Patients randomized to the PD-TR group, in addition to usual care (including their participation in a conventional physical therapy), participated in an 8-week moderate intensity AIT program involving exercise three times a week on a stationary cycle ergometer.

### 2.2. Experimental Procedures

Both groups were tested twice at the motor control laboratory of the Department of Kinesiology at the Faculty of Physiotherapy University School of Physical Education in Wroclaw (DKFP USPEW) before the eight week period of AIT (PRE) and after that time period (POST), i.e., within 6–10 days after the final AIT session, to ensure that the measurement outcome values in the PD-TR group were not due to the effects of the final single training session. The 8-week AIT program for PD-TR group was carried out in the facilities of the DKFP USPEW. The PD-CO group maintained their normal daily physical activity during this eight week time period (parallel design), receiving only usual care, including conventional physical therapy as prescribed and provided by the National Health Fund of Poland, which included activities for improving motor control, but without any kind of moderate or high intensity aerobic interval or continuous physical training (example: cycle ergometer, treadmill, or Nordic walking training). The patients were asked to keep an activity log (that was checked by investigators) to avoid changes in lifestyle during the project’s time frame. During the PRE and POST testing sessions, all PD patients underwent (i) motor control recording and analysis of performance in an anti-phase bimanual task using our custom-made device, (ii) a psychological evaluation of cognitive function, and (iii) a neurological assessment of PD motor and non-motor signs. The time of day for the two testing sessions (PRE and POST) was consistent for all tested subjects. All tested subjects were familiarized with the experimental protocol prior to participation in each testing session. During both testing sessions, all patients with PD were in their medication off-phase, i.e., the testing was carried out after withdrawal of anti-parkinsonian drugs for 12 h overnight. Since we assumed that the beneficial AIT effects on psychomotor function would be associated with neuroplasticity-induced improvement in dopaminergic function, we decided to test the patients with PD in their medication off-phase, i.e., without the dopaminergic treatment’s influence on dopamine level. However, during the intervention period (eight weeks of AIT program and usual care in PD-TR or eight weeks of usual care in PD-CO), the patients in both groups were in their medication on-phase, i.e., at least one hour after taking their normal daily dosage of anti-parkinsonian medication when they subjectively felt energetic. The medication doses were constant throughout this 8-week period and remained the same as those normally prescribed by their leading neurologist. We provided all patients with transportation to the testing sessions, and all patients from the PD-TR group were transported to each training session. The training supervisor monitored patients’ attendance to the AIT program.

### 2.3. Aerobic Interval Training Protocol

The 8-week AIT program consisted of three weekly 1 h training sessions (each consisting of a 10 min warm-up, 40 min of a moderate intensity aerobic interval exercise, and a 10 min cool-down phase at a slow voluntary speed) performed on a stationary cycle ergometer (MONARK, Ergomedic 874E, Vansbro, Sweden) that measured cadence (revolutions per minute, rpm) and power (W). During each training session, the 40-min aerobic interval exercise consisted of 8 sets of 5 min intervals, including 3 min of cycling at ≥60 rpm but preferably at 80–90 rpm (fast phase of interval) and 2 min of cycling at ≤60 rpm (slow phase of interval). The patients in the PD-TR group were encouraged to cycle faster (80–90 rpm or 30% faster than their voluntary pedaling rate) during the fast phase of interval and were rhythmically cued by a metronome and verbally encouraged by an instructor to pedal with the appropriate speeds for the fast and slow phases. The cadence values were presented to each subject in real-time on a screen as feedback to control the rpm during the different interval phases. The heart rate (HR, beats per minute, bpm) measured using a Polar system (Polar, Kempele, Finland), cadence (rpm), and power (W) were monitored and collected during each training session. The training supervisor adjusted the resistance for each patient to ensure they were cycling at their target heart rate (THR) and at an appropriate speed. PD patients cycled at 60–75% of their individualized HR_max_ (maximal heart rate), which was predicted for each patient based on the formula developed by Tanaka et al. [34]. The PD patients were encouraged to increase their THR every 2 weeks by 5% (60% of the HR_max_ during weeks 1–2, 65% during weeks 3–4, 70% during weeks 5–6, and 75% during weeks 7–8 of the training period). The averaged values of the percentage of HR_max_, cadence, and power of the eight intervals for each training session for each subject during the fast and slow phases of AIT were calculated. Then, the average value of the 24 training sessions for each parameter was calculated. Additionally, the patients’ perceptions of the exercise intensity (for the fast and slow phases of the interval) were assessed using the Borg Rating of Perceived Exertion (RPE) scale [35].

### 2.4. Primary Outcomes

#### 2.4.1. Bimanual Motor Control Recording and Analysis

**Figure 2:**
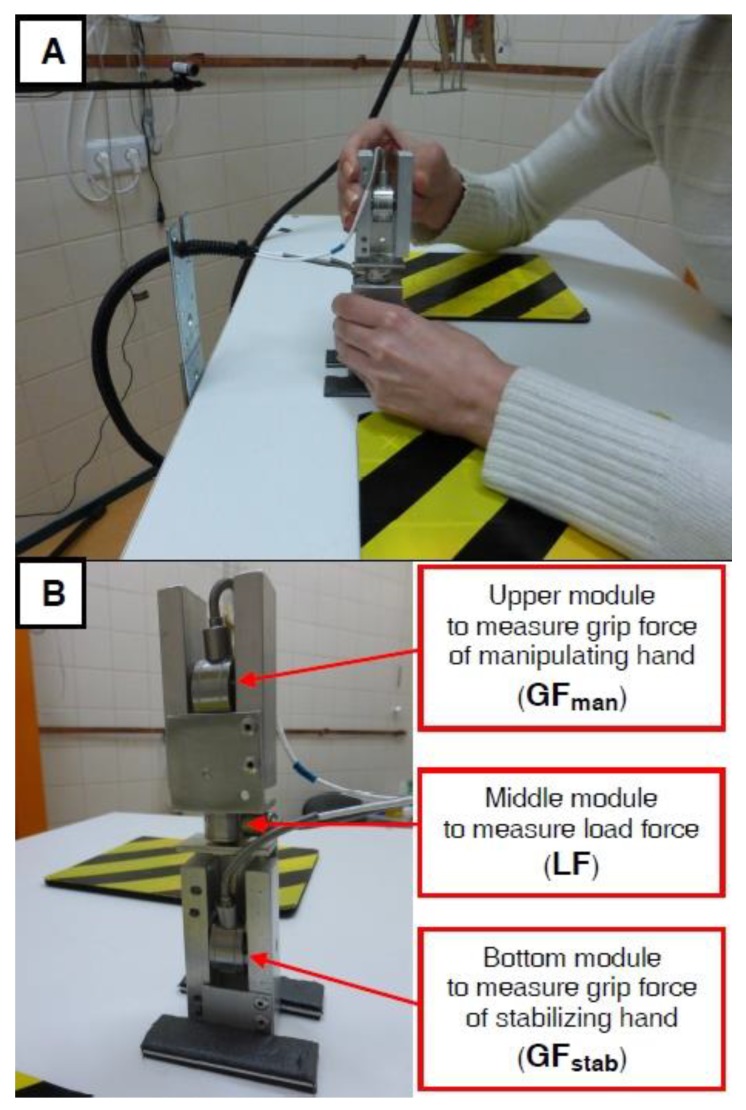
Images describing the set-up (panel **A**) and our custom-made device (panel **B**) used for the anti-phase bimanual motor control measurements.

The bimanual motor control recordings and analyses were performed by the same investigator (from the DKFP USPEW) during both testing sessions, who was not blinded to the groups tested. The PD patients were asked to perform an anti-phase bimanual motor coordination task using our custom-made device (Figure 2), which is a modification of the device used in other studies [21,36]. This motor task is an imitation of a daily life bimanual task, similar to that performed when a person opens a rectangular container of perfume by pulling (without rotation) the rectangular cap of the container with one hand (manipulating hand) while stabilizing the lower rectangular part of the container with the second hand (stabilizing hand). During the testing sessions, the patients performed this task while sitting in a chair with their forearms comfortably resting on a table, and the measurement device was placed at the midline between the two hands (Figure 2A). Our device consists of the following three modules (Figure 2B): (1) two identical modules that measure the normal grip force (GF; squeezing between the thumb and second to fifth fingers), i.e., the upper module was for the manipulating hand (GF_man_) and the bottom module was for the stabilizing hand (GF_stab_), and (2) a middle module, positioned between the upper and bottom modules, which measured the load force (LF), i.e., the extension tangential force generated by both hands pulling against each other to overcome the device’s resistance. 

The experimental motor task consisted of the following sequential motor activities performed by an instructed subject (all subjects received the same instructions) after an auditory signal: (i) moving both hands toward the measurement device, (ii) squeezing the upper module with the more affected hand and the bottom module with the less-affected hand, and (iii) generating the LF, i.e., “pulling isometrically”—pulling the upper and bottom modules in opposite directions while avoiding movement of the measurement device. Appropriate performance in the LF generation task requires synchronized automatic coordination between the hand that simultaneously squeezes and pulls the upper module (manipulating hand) and the hand that stabilizes the device by simultaneous squeezing and pulling on the lower module (stabilizing hand). Because the neural substrates of this automatic bimanual motor coordination and feed forward control to adjust GFs to LF are dopamine-dependent extra pyramidal structures (striatum) and cortical areas (pre-motor areas) [2,3,5], we hypothesized that this bimanual task paradigm could be a sensitive experimental method to indirectly assess any AIT-induced CNS improvement in our PD patients, as reported in other studies [21,36].

The subjects performed three trials of maximal isometric LF generation (separated by 3 min resting intervals) from which we calculated an averaged value of maximal voluntary contraction (MVC) of LF. After a 3 min rest, the subjects performed three trials of 20% MVC of isometric LF generation with a 2 min interval between trials. The force feedback provided to the subject (on the LCD screen) during the three 20% MVC of LF trials included the actual generated LF signal and a horizontal cursor indicating the set target of 20% MVC of LF that must be achieved during each trial. 

To assess the automatic (subconscious) adjustment control of GF at 20% MVC of LF, we primarily analyzed the following three parameters: peak value of GF in the manipulating hand (GF_man_), time taken for GF_man_ development (time GF_man_ dev), and rate of GF_man_ development (rate GF_man_ dev). The possible AIT-induced changes in the parameters describing the automatic adjustment of GF to LF might also be related to inter-group and inter-testing session differences in the ability to generate a maximal level of LF (100% LF) and consequently, to differences in the values of parameters describing the generation of 20% LF. These are the following parameters: the absolute value of LF (20% LF), and the time taken and rate of 20% LF development (time 20% LF dev and rate 20% LF dev). Therefore, to rule out any changes in LF control from the assessment of GF adjustment to LF, we additionally analyzed these load force parameters.

To assess the quality of automatic bimanual inter-limb coordination, we analyzed the delay between the onset of GF development in the manipulating hand and that in the stabilizing hand (time GFs del _man-stab_). For more details regarding the description of our custom-made device as well as the data recording, processing, and analysis, see the text in Supplementary file S1.

#### 2.4.2. Psychological Assessment of Cognitive Function

All PD patients were evaluated by an experienced psychologist (investigator from the CN MUW) who was blinded to the tested groups. The investigator primarily assessed executive function using the following two psychological tests: the Trail Making Test (TMT, parts A and B) [27,28] and the Stroop Test (ST, parts I and II) [29,30,31]. In both the TMT and ST tests, the less time required to accurately complete the test, the better the performance. The TMT-A is primarily used to examine the cognitive processing speed, and the TMT-B is used to examine executive function [27,28]. The ST-I is used as a measure of processing speed, and the ST-II is used as a measure of selective attention and inhibition [29,30,31].

### 2.5. Secondary Outcomes

#### Neurological Assessment of Parkinson’s Disease Signs

All individuals with PD were evaluated by an experienced neurologist (investigator from the CN MUW) who was blinded to the tested groups. This investigator specialized in movement disorders using the Hoehn and Yahr scale, the Unified Parkinson’s Disease Rating Scale (UPDRS), and the Schwab and England Daily Living Activity Scale [33]. We analyzed the parkinsonian bradykinesia scores of the more affected upper extremity (Brad.-UE_UPDRS 23_) of each patient based on the UPDRS’s finger tapping item (item 23). We also analyzed the performance of daily life manual functions (DLMF_UPDRS 8–11_: handwriting, cutting food, handling utensils, dressing, and hygiene as the sum of the scores of items 8–11 on the UPDRS). Finally, intellectual and emotional states (Int.Beh. _UPDRS 1–4_: intellectual impairment, thought disorder, depression, motivation/initiative as the sum of the scores on items 1–4 on the UPDRS) were measured [33]. Based on the Schwab and England Scale, we also assessed the activities of daily living (S&E DLA scale) [33].

### 2.6. Statistical Analysis

For the bimanual motor control recordings, we obtained the average value of three consecutive trials for each subject. The psychological and neurological tests were conducted once per testing session. All parameters were expressed as group mean and standard deviation values. The Shapiro–Wilk test was performed to estimate the parameters’ distribution. The Wilcoxon signed-rank test or Student’s t-test (chosen based on the Shapiro-Wilk test and the scale type applied) was performed to test the statistical significance of the differences (if any) in paired-sample comparisons (between the PRE and POST testing sessions). For the unpaired-sample comparisons (between the PD-TR and PD-CO groups), the Mann-Whitney test or Student’s t-test (as appropriate) was applied. We also calculated the Cohen effect size (*d* value) for both factors (testing session and tested group) for all parameters; a value of 0 indicated no effect, whereas values >0.2, ~0.6, and >0.8 indicated small, moderate, and large effects, respectively. The effect size was calculated as $d=\frac{M_{2}-M_{1}}{SD_{pooled}}$, where M is the mean value of the sample, and SD_pooled_ is the pooled standard deviation, which was calculated as $SD_{pooled}=\sqrt{\left(SD_{1}^{2}+SD_{2}^{2}\right)/2}$. An α value ≤0.05 was taken as statistically significant for all analyses, which were performed using SPSS Statistics 22.0 software (IBM, Armonk, NY, USA).

## 3. Results

### 3.1. Inter-Group Comparison (PD-TR vs. PD-CO) of Clinical and Anthropometric Parameters

We compared the clinical and anthropometric parameters between the PD-TR and PD-CO groups and found that there were no significant inter-group differences in these parameters (Table 1). The following *p* and *F* values were obtained for these comparisons: age (*p* = 0.483, *F* = 0.513); body mass (*p* = 0.888, *F* = 0.017); height (*p* = 0.870, *F* = 0.027); disease onset (*p* = 0.213, *F* = 1.670); disease duration (*p* = 0.389, *F* = 0.778); Hoehn and Yahr scale (*p* = 0.835, *F* = 0.045).

### 3.2. Data on Exercise Intensity and Attendance at AIT Testing Sessions

The average values for %HR_max_, cadence, and power attained in the 24 AIT sessions for the fast and slow phases were as follows: %HR_max_ 68 ± 10 vs. 62 ± 9 bpm; cadence 69 ± 13 vs. 42 ± 5 rpm, and power 38 ± 33 vs. 22 ± 19 W. The average reported RPE values for the fast phase was 18, i.e., very hard, and for the slow phase, 14, i.e., somewhat hard.

Patients from the PD-TR group attended, on average, 23.44 ± 0.73 out of the 24 AIT sessions (97.66% attendance) with complete attendance (24/24 sessions) by six patients, participation in 23/24 sessions by three patients, and participation in 22/24 sessions by one patient. All 20 patients underwent all assessments in the PRE and POST testing sessions (performed in spring: March–June), and all primary and secondary outcomes from these assessments were finally analyzed.

### 3.3. Bimanual Motor Control Outcomes

**Figure 3:**
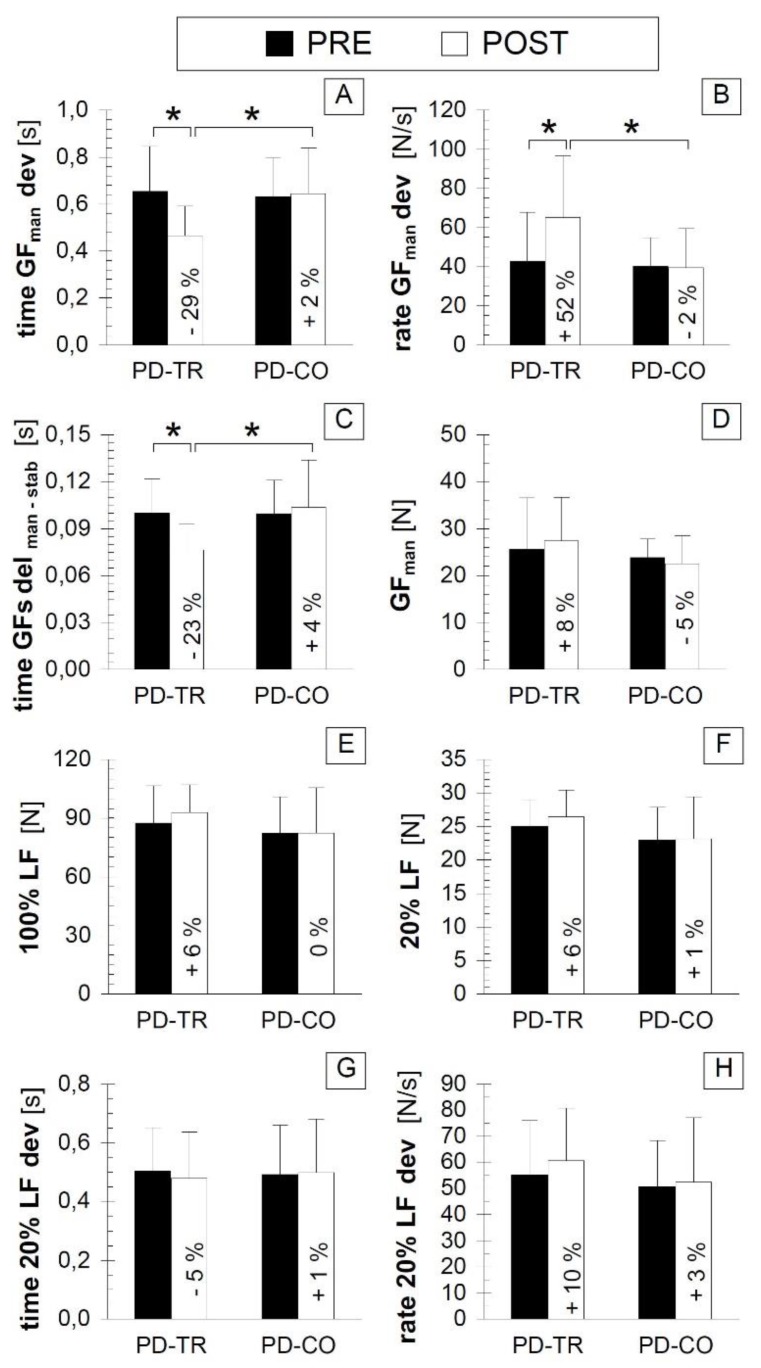
Comparison of the bimanual motor control outcomes between the two testing sessions and the two tested groups. (**A**) panel A presenting the values of time GFman dev; (**B**) panel B presenting the values of rate GFman dev; (**C**) panel C presenting the values of time GFs del _man-stab_; (**D**) panel D presenting the values of GFman; (**E**) panel E presenting the values of 100% LF; (**F**) panel F presenting the values of 20% LF; (**G**) panel G presenting the values of time 20% LF dev; (**H**) panel H presenting the values of rate 20% LF dev. Abbreviations: PRE—testing session before the eight weeks of training; POST—testing session after the eight weeks of training; PD-TR—Parkinson’s disease patients from the training group; PD-CO—Parkinson’s disease patients from the control group; GFman—peak value of grip force in the manipulating hand during the 20% load force trial; time GFman dev—time taken for GFman development; rate GFman dev—rate of GFman development; time GFs del _man-stab_—time delay between the onset of grip force development in the manipulating hand and that in the stabilizing hand; 100% LF—the value of achieved maximal load force; 20% LF—the peak value of developed LF during the trial of 20% LF; time 20% LF dev—the time taken for 20% LF development; rate 20% LF dev—the rate of 20% LF development; the % value inserted into the bar of the POST session indicates the percentage value of the change between the POST and PRE testing sessions; *—statistically significant inter-testing session or inter-group difference at the *p* ≤ 0.05 level.

A significant improvement was observed in the POST vs. PRE comparison in the PD-TR group for the automatic adjustment of GF to generate LF, as reflected by the shortened time and increased rate of GF development in the manipulating hand (time GFman dev and rate GFman dev, respectively) (Figure 3A,B; *p* < 0.05, Cohen’s d value indicated large and moderate effect sizes, respectively, Table 2). No inter-session differences were observed in the PD-CO group for these parameters (Figure 3A,B; *p* > 0.05, Table 2).

Additionally, in the PD-TR group, in the POST vs. PRE comparison, we noted an improvement in automatic inter-limb coordination, as reflected by the shortened time of delay between the initiation of the grip force development in the manipulating hand and that in the stabilizing hand (GFs del _man-stab_) (Figure 3C; *p* < 0.05, Cohen’s d value indicated a large effect size, Table 2), and no inter-session differences were observed in the PD-CO group (Figure 3C; *p* > 0.05, Table 2). The values of time GFman dev, rate GFman dev, and GFs del _man-stab_ (Figure 3A–C, respectively) did not differ between the PD-TR and PD-CO groups during the PRE testing (*p* > 0.05, Table 2) but worsened in the PD-CO group compared to the PD-TR group during the POST testing session (*p* < 0.05, Cohen’s d value indicated a large effect size, Table 2). 

Importantly, the additionally analyzed parameters, i.e., the GFman, 100% LF, 20% LF, time 20% LF dev, and rate 20% LF dev did not differ between the PRE and POST testing sessions, either in the PD-TR group or in the PD-CO group (*p* > 0.05) and did not differ between the PD-TR and PD-CO groups during either the PRE or POST testing sessions (*p* > 0.05) (Figure 3D–H; Table 2).

### 3.4. Psychological Cognitive Outcomes

**Figure 4:**
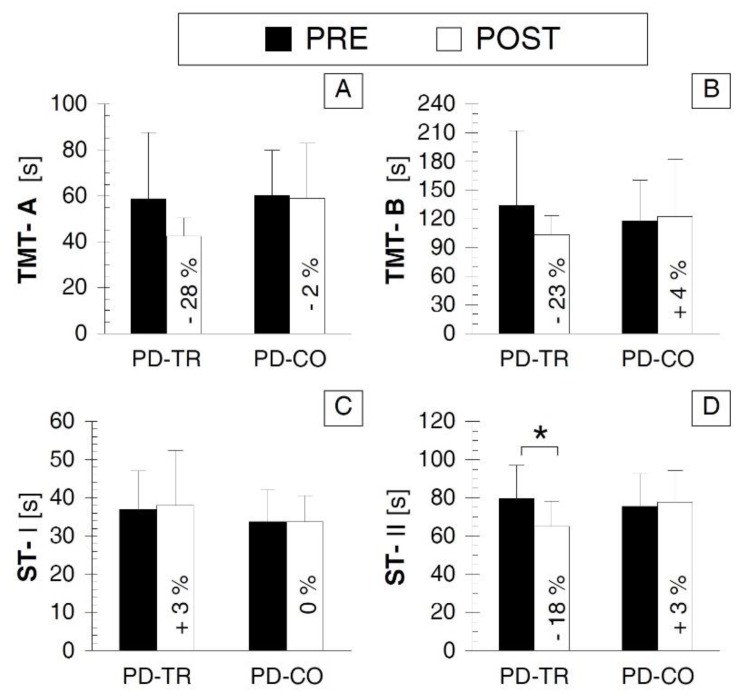
Comparison of psychological cognitive outcomes between the two testing sessions and the two tested groups. (**A**) panel A presenting the values of TMT-A; (**B**) panel B presenting the values of TMT-B; (**C**) panel C presenting the values of ST-I; (**D**) panel D presenting the values of ST-II. Abbreviations: PRE—testing session before the eight weeks of training; POST—testing session after the eight weeks of training; PD-TR—Parkinson’s disease patients from the training group; PD-CO—Parkinson’s disease patients from the control group; TMT-A—part A of the Trail Making Test; TMT-B—part B of the Trail Making Test; ST-I—part I of the Stroop Test; ST-II—part II of the Stroop Test; *—statistically significant inter-testing session or inter-group difference at the *p* ≤ 0.05 level; the % value inserted into the bar of the POST session indicates the percentage value of the change between the POST and PRE testing sessions.

The ST-II values (Figure 4) were significantly improved in the PD-TR group in the POST vs. PRE comparison (*p* < 0.05, Cohen’s d value indicated a large effect size, Table 2) but did not change in the PD-CO group (*p* > 0.05, Table 2) and did not differ significantly between the PD-TR and PD-CO groups during either the PRE or POST testing sessions (*p* > 0.05, Table 2). However, we would like to underline that there was a clear tendency towards a shorter time taken for ST-II performance in the PD-TR vs. PD-CO in the POST testing session; namely, there was a high Cohen’s “d” value (d = 0.853) showing a large effect size for this comparison and a *p* value close to the α ≤ 0.05 level (*p* = 0.073).

No difference was observed in the TMT-A, TMT-B, or ST-I values between the PRE and POST testing sessions in either the PD-TR group or the PD-CO group (*p* > 0.05) or between the PD-TR and PD-CO groups during the PRE or the POST testing session (*p* > 0.05) (Figure 4A–C; Table 2). However, notably, a clear tendency toward lower values at POST testing compared with at PRE testing was observed for the TMT-A and TMT-B values in the PD-TR group (Figure 4A,B; Table 2).

### 3.5. Neurological Outcomes

**Figure 5:**
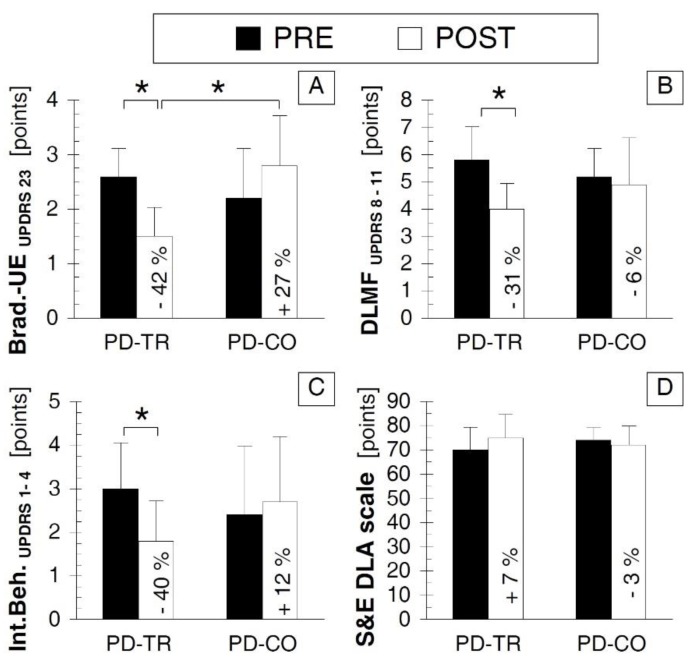
Comparison of neurological outcomes between the two testing sessions and two tested groups. (**A**) panel A presenting the values of Brad.-UE_UPDRS 23_; (**B**) panel B presenting the values of DLMF_UPDRS 8–11_; (**C**) panel C presenting the values of Int.Beh. _UPDRS 1–4_; (**D**) panel D presenting the values of S&E DLA scale. Abbreviations: PRE—testing session before the eight weeks of training; POST—testing session after the eight weeks of training; PD-TR—Parkinson’s disease patients from the training group; PD-CO—Parkinson’s disease patients from the control group; UPDRS—Unified Parkinson’s Disease Rating Scale; Brad.-UE_UPDRS 23_—bradykinesia of the upper extremity based on point 23 from the UPDRS; DLMF_UPDRS 8–11_—daily life manual functions based on the sum of points 8–11 from the UPDRS; Int.Beh. _UPDRS 1–4_—intellectual and emotional state based on the sum of points 1–4 from the UPDRS; S&E DLA scale—Schwab and England Daily Living Activity Scale; *—statistically significant inter-testing session or inter-group difference at the *p* ≤ 0.05 level; the % value inserted into the bar of the POST session indicates the percentage value of the change between the POST and PRE testing sessions.

The Brad.-UE_UPDRS 23_ (Figure 5A) was improved in the PD-TR group in the POST vs. PRE comparison (*p* < 0.05, Cohen’s d value indicated a large effect size, Table 2) but did not significantly change in the PD-CO group (*p* > 0.05, Table 2) and did not differ between the PD-TR and PD-CO groups during PRE testing (*p* > 0.05, Table 2) but was lower in the PD-TR group than in the PD-CO group during POST testing (*p* < 0.05, Cohen’s d value indicated a large effect size, Table 2). The DLMF_UPDRS 8–11_ and Int.Beh. _UPDRS 1–4_ (Figure 5B,C, respectively) were improved according to the POST vs. PRE comparison (*p* < 0.05, Cohen’s d value indicated a large effect size, Table 2) in the PD-TR group but did not change in the PD-CO group (*p* > 0.05, Table 2) and did not differ between the PD-TR and PD-CO groups during either the PRE or POST testing sessions (*p* > 0.05, Table 2). The S&E DLA scale value (Figure 5D) did not differ between the PRE and POST testing sessions either in the PD-TR group or in the PD-CO group (*p* > 0.05, Table 2) and did not differ between the PD-TR and PD-CO groups during the PRE or POST testing sessions (*p* > 0.05, Table 2).

## 4. Discussion

Our results show that following the 8-week moderate intensity AIT program, individuals with PD exhibited improved psychomotor behaviors, reflected by their bimanual motor control, executive function, and neurological symptoms evaluation. Our findings provide further evidence for the benefits of exercise but additionally introduce moderate intensity aerobic interval training regimen on the cycle ergometer as a promising physical training modality for PD patients.

### 4.1. Why AIT May Be An Advantageous Treatment Approach for PD

Previously published human studies [14,16,17,21,22] demonstrated the beneficial effects of continuous intensive training protocols on the motor and non-motor aspects of Parkinson’s disease, but the current study is the first randomized controlled study to present a positive generalized effect with a moderate intensity aerobic interval training (AIT) regimen on a cycle ergometer. Alberts et al. [36] showed that high intensity aerobic continuous pedaling on a tandem bicycle causes a hemodynamic effect in the basal ganglia (evaluated with functional magnetic resonance imaging) in trained PD patients. Like the continuous regimen reported by Alberts et al. [36], our moderate intensity AIT may have caused greater blood flux into the basal ganglia as well. This greater blood flux in the basal ganglia may have led to BDNF release by the vascular endothelium, since the endothelial cells secrete BDNF as a response to blood shear stress in the vessels (Prigent-Tessier et al. [37]). Previous studies [24,25,26] have established that interval training is more enjoyable and effective than continuous exercise programs for non-PD populations. Because the goal is to motivate individuals with PD to adopt a lifelong commitment to exercise, enjoyment is an important factor for improving adherence to exercise [24]. Importantly, other studies have shown that the BDNF increase following interval training is greater than that following continuous regimen training [38,39]. Increased levels of BDNF facilitate neuroplasticity-related motor and cognitive improvements in PD patients, as higher levels of neurotrophic factors (including BDNF) in the basal ganglia have known cytoprotective effects on dopamine-dependent neural structures [40,41,42]. Importantly, we previously demonstrated increased blood BDNF levels after eight weeks of moderate intensity AIT in PD patients [20,23].

### 4.2. AIT-Induced Improvement in Bimanual Motor Control

We found an AIT-induced improvement in the adjustment of grip force to load force in the manipulating hand and in the inter-limb coordination during the performance of a bimanual anti-phase motor function task in the PD-TR group. Importantly, in terms of the additionally analyzed parameters, i.e., the GFman, 100% LF, 20% LF, time 20% LF dev and rate 20% LF dev, we did not find any inter-testing session or inter-group differences, which strengthens our interpretation and conclusion regarding the AIT-induced improvement of subconscious (automatic) GF *adjustment* motor control in the manipulating hand during the conscious performance of bimanual LF generation in the PD-TR group. Other studies [21,36] reported similar improvements (with large effect sizes based on a Cohen’s d value of −1.59) in bimanual dexterity in individuals with PD who participated in continuous forced intensive pedaling on a tandem bicycle. In their studies, the individuals with PD performed eight weeks (three times a week) of a pedaling regime similar to the one used in our study (80–90 rpm or 30% faster than their volitional speed). Importantly, anti-phase bimanual performance has been identified as a PD-related deficit [2,3,4,5,6]. The neural substrates of bimanual motor coordination and feed forward control to adjust grip force to load force are dopamine-dependent extra pyramidal structures (striatum) and cortical areas (pre-motor areas) [2,3,4,5]. In our study, the AIT-induced changes in the subconscious (automatic) adjustment of GF during the conscious performance of the bimanual LF generation task are likely related to an improvement in the functioning of this extra pyramidal system, which is responsible for subconscious motor task performance and bimanual coordination.

### 4.3. AIT-Induced Improvement in Executive Function

Our finding of AIT-induced improvement in executive function is consistent with the results reported by Duchesne et al. [22], who showed an increase in Stroop test performance following aerobic exercise. The clear tendency for improvement in other cognitive measures (reflected in the Trail Making Test) in the PD-TR group is consistent with the results of Ridgel al. [43] who found a similar improvement as an effect of passive lower extremity movement during training on a tandem bicycle in PD patients and as a reaction to regular Nordic walking in healthy elderly people [44]. Ridgel et al. [43] suggested that such exercise might cause an increase in cerebral blood flow, leading to neuroplastic changes in the CNS and consequently, improved cognitive function in PD patients. Additionally, the results of Tabak et al. [17] support the psychological results of our study, because these authors found that eight weeks of continuous intensive exercise on a cycle ergometer led to improvements in cognitive measures in PD patients. The cognitive functions tested in the present study, namely executive and inhibitory function, depend on the integrity of dopaminergic subcortical and cortical interconnections, such as the connections between the substantia nigra and the striatum, the striatum and the supplementary motor area (SMA), and the striatum and the dorsolateral prefrontal cortex.

### 4.4. AIT-Induced Improvement in Motor and Non-Motor Signs of PD

Our study demonstrated an amelioration of upper extremity bradykinesia and an improvement in daily life manual functions, and mood and intellectual functions in the PD-TR group. This is consistent with results reported by other researchers [19,21,23,36], who found that intensive aerobic exercise on a tandem or standard cycle ergometer improved motor symptoms in PD. It has been suggested that this improvement is related to an exercise-induced increase in BDNF levels in trained patients. Furthermore, the improvement in activities of daily living, mood, emotional state, cognitive function, and overall quality of life with exercise [45] may reflect exercise-induced neuroplastic changes in the CNS. Marusiak et al. [23] reported that an alleviation of parkinsonian rigidity and decreased resting muscle stiffness (measured with myometry) were correlated with an increase in BDNF levels in patients with PD who participated in moderate intensity AIT [23]. In the present study, we found a generalized beneficial effect of moderate intensity AIT on the DA-dependent motor and non-motor signs that are compromised in PD patients [1].

### 4.5. Limitations and Implications of Our Findings

In the present study, we evaluated the generalized effects of moderate intensity AIT on psychomotor behaviors and did not measure the CNS dopaminergic function changes that we considered in our discussion. However, the outcome measures that were chosen were specific to PD-related psychomotor tasks that engage dopamine-dependent cortical and subcortical structures [2,3,5]. The beneficial impact of an AIT program on psychomotor behavior in PD provides a basis for the use of AIT as a treatment approach in the overall therapeutic strategy. Better cognitive and motor function may consequently be associated with a milder disease stage and patient ambulatory status [46], which is important from patient quality of life and treatment cost points of view [47]. Thus, our understanding of mechanisms of the influence of AIT on psychomotor function in PD patients has practical relevance. However, to understand the exact neurophysiological mechanisms of these AIT-induced behavioral benefits (psychomotor function improvement), more research using a variety of neuroimaging methods (electroencephalography, functional magnetic resonance imaging, and positron emission tomography) with a bigger sample size (to increase statistical power and findings generalizability) needs to be conducted. 

An important limitation of this study is the relatively small sample size. Nevertheless, the appropriate statistical analysis applied by us was able to reveal the effects of AIT on outcome measures even in this relatively small sample size. However, the generalized effects of AIT on psychomotor function in trained patients with PD that we observed based on present comparative analyses warrant further studies with larger sample sizes to allow for more discerning analyses. These future studies will provide evidence-based knowledge about the benefits of moderate intensity AIT that can be generalized for the whole PD patient population and will be a base for implementation of this kind of exercise as a prescribed treatment form that is recommended and funded by the National Health Fund.

## 5. Conclusions

Eight weeks of moderate intensity aerobic interval training by individuals with Parkinson’s disease improved psychomotor behaviors, as reflected by bimanual motor control, executive function, and neurological signs of PD.

## Figures and Tables

**Table 1:** Clinical and anthropometric characteristics of the Parkinson’s disease (PD) patients in the training (PD-TR, *n* = 10) and control (PD-CO, *n* = 10) groups.

PD Patient No.	Age (Years)	Sex (F/M)	Body Mass (kg)	Height (cm)	Disease Onset/Duration (Years)	Affected Upper Limb (R/L)	Dominant Upper Limb (R/L)	Hoehn and Yahr (Points)
PD-TR#01	79	M	71	157	69/10	R	R	2.5
PD-TR#02	65	F	64	162	58/7	L	R	2.5
PD-TR#03	68	M	78	167	55/13	R	R	2.5
PD-TR#04	65	F	61	155	63/2	R	R	1.5
PD-TR#05	60	M	79	169	46/14	R	R	3.0
PD-TR#06	84	M	73	165	80/4	L	R	1.5
PD-TR#07	68	M	67	177	56/12	L	R	2.5
PD-TR#08	78	F	67	159	70/8	L	R	3.0
PD-TR#09	62	M	63	161	46/16	R	R	3.0
PD-TR#10	88	F	67	149	84/4	L	R	2.0
Mean ± SD	72 ± 10	-	69 ± 9	162 ± 8	63 ± 13/9 ± 5	-	-	2.35 ± 0.57
PD-CO#01	81	F	63	159	71/10	L	R	3.0
PD-CO#02	67	F	64	156	63/4	R	R	2.0
PD-CO#03	81	M	67	157	59/12	R	R	2.0
PD-CO#04	90	F	60	148	84/6	L	R	2.0
PD-CO#05	79	F	60	156	69/8	L	R	2.5
PD-CO#06	72	M	78	182	61/11	R	R	2.0
PD-CO#07	60	F	67	153	52/8	R	R	2.0
PD-CO#08	74	F	59	164	69/5	R	R	1.5
PD-CO#09	66	F	70	160	64/2	L	R	1.5
PD-CO#10	70	M	76	166	55/15	R	R	3.0
Mean ± SD	74 ± 9	-	66 ± 7	160 ± 9	66 ± 9/8 ± 4	-	-	2.25 ± 0.53

Abbreviations: No.—number; F—female; M—male; R—right side; L—left side; ±SD—standard deviation.

**Table 2:** Significance (*p*) and effect size (d) values for the parameter comparisons between the testing sessions (POST vs. PRE) and the tested groups (PD-TR, *n* = 10 vs. PD-CO, *n* = 10).

Parameters		POST vs. PRE Comparison		PD-TR vs. PD-CO Comparison	
		PD-TR	PD-CO	PRE	POST
Bimanual Motor Control Outcomes	time GFman dev	*p* = 0.013 * d = –1.161	*p* = 0.508 d = 0.065	*p* = 0.821 d = −0.113	*p* = 0.025 * d = 1.097
	rate GFman dev	*p* = 0.013 * d = 0.781	*p* = 0.816 d = −0.051	*p* = 0.793 d = −0.119	*p* = 0.044 * d = −0.970
	time GFs del _man-stab_	*p* = 0.020 * d = –1.183	*p* = 0.592 d = 0.152	*p* = 0.993 d = −0.004	*p* = 0.023 * d = 1.108
	GFman	*p* = 0.148 d = 0.189	*p* = 0.288 d = −0.252	*p* = 0.646 d = −0.209	*p* = 0.173 d = −0.635
	100% LF	*p* = 0.232 d = 0.304	*p* = 0.993 d = 0.001	*p* = 0.534 d = −0.284	*p* = 0.246 d = −0.536
	20% LF	*p* = 0.275 d = 0.357	*p* = 0.213 d = 0.026	*p* = 0.330 d = −0.448	*p* = 0.186 d = −0.619
	time 20% LF dev	*p* = 0.449 d = −0.153	*p* = 0.725 d = 0.034	*p* = 0.880 d = −0.068	*p* = 0.809 d = 0.110
	rate 20% LF dev	*p* = 0.123 d = 0.269	*p* = 0.645 d = 0.076	*p* = 0.705 d = −0.223	*p* = 0.424 d = −0.365
Psychological Outcomes	TMT-A	*p* = 0.083 d = −0.767	*p* = 0.838 d = −0.055	*p* = 0.650 d = 0.057	*p* = 0.055 d = 0.918
	TMT-B	*p* = 0.216 d = −0.553	*p* = 0.722 d = 0.082	*p* = 0.558 d = −0.267	*p* = 0.359 d = 0.421
	ST-I	*p* = 0.751 d = 0.097	*p* = 0.566 d = 0.000	*p* = 0.363 d = −0.336	*p* = 0.401 d = −0.385
	ST-II	*p* = 0.007 * d = –0.953	*p* = 0.140 d = 0.136	*p* = 0.593 d = −0.244	*p* = 0.073 d = 0.853
Neurological Outcomes	Brad.-UE_UPDRS 23_	*p* = 0.015 * d = –2.108	*p* = 0.107 d = 0.653	*p* = 0.354 d = −0.537	*p* = 0.003 * d = 1.735
	DLMF_UPDRS 8–11_	*p* = 0.004 * d = –1.643	*p* = 0.616 d = 0.211	*p* = 0.242 d = −0.528	*p* = 0.309 d = 0.646
	Int.Beh. _UPDRS 1–4_	*p* = 0.005 * d = –1.214	*p* = 0.279 d = 0.195	*p* = 0.331 d = −0.447	*p* = 0.122 d = 0.726
	S&E DLA	*p* = 0.059 d = 0.522	*p* = 0.317 d = −0.300	*p* = 0.155 d = 0.526	*p* = 0.419 d = −0.339

Abbreviations: PRE—testing session before the eight weeks of training; POST—testing session after the eight weeks of training; PD-TR—Parkinson’s disease patients from the training group; PD-CO—Parkinson’s disease patients from the control group; GFman—peak value of grip force in the manipulating hand during the 20% load force trial; time GFman dev—time taken for GFman development; rate GFman dev—rate of GFman development; time GFs del _man-stab_—time delay between the onset of GF development in the manipulating hand and that in the stabilizing hand; 100% LF—the achieved maximal value of load force; 20% LF—the peak value of developed LF during the 20% LF trial; time 20% LF dev—the time taken for 20% LF development; rate 20% LF dev—the rate of 20% LF development; TMT-A—part A of the Trail Making Test; TMT-B—part B of the Trail Making Test; ST-I—part I of the Stroop Test; ST-II—part II of the Stroop Test; UPDRS—Unified Parkinson’s Disease Rating Scale; Brad.-UE_UPDRS 23_—bradykinesia of the upper extremity based on point 23 from the UPDRS; DLMF_UPDRS 8–11_—daily life manual functions based on the sum of points 8–11 from the UPDRS; Int.Beh. _UPDRS 1–4_—intellectual and emotional state based on the sum of points 1–4 from the UPDRS. S&E DLA scale—Schwab and England Daily Living Activity Scale; *p*—significance value of the inter-testing session or inter-group comparison; d—the Cohen effect size value for the both factors (testing session and tested group); a value of 0 indicated no effect, whereas values >0.2, ~0.6, and >0.8 indicated small, moderate, and large effects, respectively; * statistically significant inter-testing session or inter-group difference at the *p* ≤ 0.05 level.

## Supplementary Materials

The following are available online at https://www.mdpi.com/1660-4601/16/5/880/s1. Supplementary File 1: Supplementary detailed description of bimanual motor control assessment using our custom-made device, and descriptions of data recording, processing, and analysis.
