# Supplementary material for: Eight Weeks of Aerobic Interval Training Improves Psychomotor Function in Patients with Parkinson’s Disease—Randomized Controlled Trial
Source: Int J Environ Res Public Health. 2019 Mar 11;16(5):880. doi: 10.3390/ijerph16050880 (PMC6427316; doi:10.3390/ijerph16050880)
Supplement: Supplementary file 1 [file ijerph-16-00880-s001.pdf]

S1 File. Supplementary detailed description of bimanual motor control assessment using our custom-made device, and the description of data recording, processing, and analysis.

Our device consists of the following three modules (Figure 2B): (1) two identical modules that measure the normal grip force (GF; squeezing between the thumb and second to fifth fingers), i.e., the upper module for the manipulating hand (GFman) and the bottom module for the stabilizing hand (GFstab), and (2) an in-built middle module between the upper and bottom modules that measures the load force (LF), i.e., the extension tangential force generated by both hands pulling against each other to overcome the device's resistance. The upper and bottom modules comprise two small metal plates with a tension/extension force transducer between the plates (Figure 2B). The middle module also has a tension/extension transducer between the upper and bottom modules (Figure 2B). Due to the construction of the measurement device, the device is stiff and inseparable, enabling the measurement of GF and LF isometrically. The force transducers (WMC, Interface, Arizona, USA) used in our device are highly sensitive to the levels of generated GF and LF (force measurement range: for the upper and bottom modules 0–220 N, and for the middle module 0–120 N). The output signals of the transducers were filtered, amplified (with SGA conditioners, Interface, Arizona, USA), and then directed to a multichannel CED card (Cambridge Electronic Design, Ltd., Cambridge, UK) and recorded using Spike 2 software (version 7.10, Cambridge Electronic Design, Ltd., Cambridge, UK). The sampling frequency of simultaneously recorded force signals was 2048 Hz.

The motor task required the subjects to respond to an auditory cue by (i) moving both hands to the measurement device, (ii) squeezing the upper module with the more affected hand and the bottom module with the less-affected hand, and (iii) generating the LF, i.e., "pulling isometrically"—pulling the upper and bottom modules in opposite directions while avoiding movement of the measurement device. The subjects were asked

to perform three trials of maximal isometric LF generation, separated by 3 min rest intervals. The maximal voluntary contraction (MVC) of LF was then obtained as an average value calculated from the three trials. After a 3 min rest, the subjects performed three trials of 20% MVC of isometric LF generation with 2 min intervals between trials. All trials of LF generation at 100% and 20% of MVC were performed with the instruction to develop the force as soon as possible. Force feedback was provided during the performance of the three LF trials at the level of 20% MVC and included (i) the actual generated LF signal and (ii) a horizontal cursor indicating the set target of 20% MVC of LF that must be achieved in each trial.

Based on the signal processing algorithms implemented in the CED Spike2 environment, we automatically detected (i) the onset of LF and GF generation development (as the time of the occurrence of a force value equal to three standard deviations from the baseline force signal) and (ii) the time and values of the peak LF and peak GFs during the trials of 100% and 20% of maximal LF generation. Based on the values of the above-mentioned parameters, we calculated (iii) the rate of LF and GF development as the quotient of the developed peaks of the LF and GF values to the time taken for this force development during the 20% MVC LF trials, and (iv) the grip time delay as the time interval between the onset of GF in the manipulating hand and that in the stabilizing hand (during the 20% MVC LF trials).

To assess the control of GF to 20% MVC of LF, we primarily analyzed the following three parameters: peak value of GF in the manipulating hand (GFman), time taken for GFman development (time GFman dev) and rate of GFman development (rate GFman dev).

The possible AIT-induced changes in the adjustment of GF to LF (based on the GFman, time GFman dev, and rate GFman dev) might also be related to inter-group and inter-testing session differences in the ability to generate a maximal level of LF (100% LF) and

consequently, to differences in the values of parameters describing the generation of 20% LF. These are the following parameters: the absolute value of LF (20% LF), the time taken and rate of 20% LF development (time 20% LF dev and rate 20% LF dev). Therefore, to rule out any changes in LF control from the assessment of GF adjustment to LF, we additionally analyzed the above-mentioned load force parameters: 100% LF, 20% LF, time 20% LF dev, and rate 20% LF dev. To assess the quality of bimanual inter-limb coordination, we analyzed the delay between the onset of GF development in the manipulating hand and that in the stabilizing hand (time GFs del man-stab).
